# Age-related reduction in anxiety and neural encoding of negative emotional memory

**DOI:** 10.3389/fnagi.2024.1375435

**Published:** 2024-07-03

**Authors:** Shefali Chaudhary, Sheng Zhang, Yu Chen, Jacqueline C. Dominguez, Herta H. Chao, Chiang-Shan R. Li

**Affiliations:** ^1^Department of Psychiatry, Yale University School of Medicine, New Haven, CT, United States; ^2^St. Luke’s Medical Center, Quezon City, Metro Manila, Philippines; ^3^VA Connecticut Healthcare System, West Haven, CT, United States; ^4^Department of Medicine, Yale University School of Medicine, New Haven, CT, United States; ^5^Department of Neuroscience, Yale University School of Medicine, New Haven, CT, United States; ^6^Wu Tsai Institute, Yale University, New Haven, CT, United States

**Keywords:** aging, anxiety, negative emotion, memory, fMRI

## Abstract

**Introduction:**

Older adults experience less anxiety. We examined how memory of negative emotional images varied with age and may reflect age-related differences in anxiety.

**Methods:**

Fifty-one adults, age 22-80 years, underwent imaging with a memory task where negative and neutral images were displayed pseudo-randomly. They were queried post-scan about the images inter-mixed with an equal number of images never displayed. Sensitivity (*d’*) and reporting bias (Z-score of false alarm rate; Z[FAR]) were quantified with signal detection theory.

**Results:**

Age was negatively correlated with both Spielberg State Trait Anxiety Inventory (STAI) state score and *d’* (negative – neutral) and positively with Z[FAR] (negative – neutral). However, STAI score and *d’* or Z[FAR] (negative – neutral) were not significantly correlated. In whole-brain regression, STAI score was correlated with higher activity of the right middle/superior temporal gyri/temporal parietal junction (MTG/STG/TPJ) for “negative correct – incorrect” – “neutral correct – incorrect” trials. Further, the MTG/STG/TPJ activity (β) was also negatively correlated with age. Mediation analyses supported a complete mediation model of age → less anxiety → less MTG/STG/TPJ β.

**Discussion:**

Together, the findings demonstrated age-related changes in negative emotional memory and how age-related reduction in anxiety is reflected in diminished temporoparietal cortical activities during encoding of negative emotional memory.

## 1 Introduction

Old versus younger adults focus more on positive than negative stimuli in emotion processing – an age-related positivity effect (Mather and Carstensen, 2005). For instance, older adults exhibited an attentional bias away from negative emotional faces in a dot probe task, while younger adults did not present such a bias (Mather and Carstensen, 2003). Similarly, in another study using dot-probe task, older and young adults were more responsive to training to direct their gaze toward positive and negative emotional pictures, respectively (Isaacowitz and Choi, 2011). Older adults in a “bad” mood looked towards positive and away from negative faces, while younger adults demonstrated mood-congruent gaze (Isaacowitz et al., 2008). Confirmed in a meta-analysis of 100 studies (Reed et al., 2014), the positivity effect is often interpreted within the socioemotional selectivity theory where shorter time horizon prompts a shift towards positive emotions as one ages.

Further, emotion influences memory, an effect that may vary across the lifespan. As one grows older, emotion becomes relatively more salient (Carstensen and Turk-Charles, 1994) and older adults appear to encode and recall emotion-laden information as well as their younger counterparts, despite an overall decline in cognitive capacity (Mather, 2004), likely because the core emotion processing circuit suffer less age-related changes (Mather, 2016).

Considering age-related positivity in emotional memory, an earlier study demonstrated evidence in support of positivity (Charles et al., 2003). However, other studies showed that emotionally negative as compared to positive events are remembered in greater contextual details in both young and old, and the accuracy of recall did not vary with age (Kensinger et al., 2002, 2007a). In a more recent work, Stam and colleagues demonstrated enhanced memory for negative (angry) versus neutral faces in young but not older adults, with the latter showing shorter eye fixation on angry versus neutral faces, possibly indicating avoidance of negative emotional stimuli (Stam et al., 2022). In another study, young and older adults had similarly enhanced memory for fearful relative to neutral faces and consistent amygdala and hippocampus activations, though young and older adults showed higher activations in the right amygdala/bilateral hippocampus and in the insula/prefrontal cortices, respectively, during fearful versus neutral face encoding (Fischer et al., 2010). Kensinger and Schacter noted poor recognition memory in older than young adults for both the negative and neutral images (Kensinger and Schacter, 2008). However, both age groups exhibited better recognition for negative versus neutral images (Kensinger and Schacter, 2008).

Imaging studies likewise presented less than consistent results. For instance, the aforementioned study noted no age differences in the neural correlates of negative versus neutral images encoding (Kensinger and Schacter, 2008). Both young and older adults showed ventromedial and dorsomedial prefrontal and orbitofrontal cortex as well as hippocampus activation during the encoding of negative or positive versus neutral pictures (Addis et al., 2010). However, collapsing across emotional and neutral pictures, older and young adults each exhibited more pronounced frontal and temporal cortical activity (Addis et al., 2010). Another study noted greater negative versus neutral picture memory in both young and older adults, but overall reduced memory enhancement of regional activities for negative versus neutral pictures recalled in older versus young adults (St Jacques et al., 2009). The latter study also noted differential activity during encoding of negative versus neutral pictures between the age groups – young versus older adults had greater activity in superior/middle frontal gyrus, hippocampus/parahippocampal gyrus, visual cortex and left amygdala, whereas older versus young adults had greater activity in dorsolateral prefrontal cortex and right amygdala (St Jacques et al., 2009). Thus, the extant findings appear to vary in the effects of age on emotional memory and the underlying neural processes, perhaps because of the characteristics of the emotional stimuli (e.g., face vs. non-face) and demand (e.g., instruction about the need to recall), of the memory task (Mather and Carstensen, 2005). Here, we revisit the issue of positivity effect and assessed potential age-related differences in the neural correlates of emotional memory.

A state of heightened worry or fear, anxiety can impact the way individuals remember negative information. For instance, individuals with higher anxiety demonstrated better performance in recalling threatening relative to non-threatening words, and the difference was associated with state anxiety scores (Reidy and Richards, 1997). Words of negative versus positive emotions or learned in negative versus neutral emotional context appeared better remembered in individuals of high versus those of low anxiety (Lee and Fernandes, 2018; Yu et al., 2018; Hakamata et al., 2022). Individuals with general anxiety disorder compared to controls had less activation in the middle cingulate gyrus and precentral gyrus during negative word encoding (Park et al., 2022). In monozygotic twins of high versus low risk for anxiety, encoding of correctly recognized negative words versus baseline led to enhanced amygdala and occipital cortical, and diminished hippocampal activity (Wolfensberger et al., 2008). Thus, anxiety may impact encoding of emotional stimuli. However, it is not clear how anxiety affects age-related variations in emotional memory encoding. For instance, older adults may show a general positivity bias in memory, while anxiety could potentially modulate and/or reflect this bias, with accompanying neural processes of emotion memory.

Here we tested these effects in 51 participants 22–80 years of age. Participants were engaged in a memory task where emotional and neutral stimuli were displayed pseudo-randomly during functional magnetic resonance imaging (fMRI) and queried post-fMRI whether they remembered seeing the stimuli presented during imaging along with a same number of new stimuli never presented. Individuals’ anxiety was assessed using the State–Trait Anxiety Inventory (STAI) (Spielberger, 1989). We calculated encoding sensitivity according to signal detection theory (SDT) as correct recognition controlling for false-positive or bias, the tendency in identifying a stimulus as present that was actually not presented (Yu et al., 2018). According to the age-related positivity effect, we hypothesized diminishing sensitivity in encoding negative relative to neutral emotional stimuli with older age and explored the corresponding age-related neural correlates during encoding. Next, we would also examine the neural processes of individual anxiety during emotion memory encoding and identified shared correlates of age and anxiety. With the shared correlates, we would employ mediation analyses to examine how age, anxiety, and the neural correlates of negative emotional memory were inter-related. As the participants did not undergo fMRI while they were queried about the images, the current study could not address the neural correlates of memory recall or of inter-subject variation in reporting bias.

## 2 Participants and methods

### 2.1 Participants and clinical assessments

We determined the sample size based on previous studies that assessed the effects of aging on emotional memory encoding, with *n* = 30–37 participants (Kensinger and Schacter, 2008; St Jacques et al., 2009; Addis et al., 2010). We screened 70 volunteers from the greater New Haven, Connecticut area. The inclusion/exclusion criteria included (1) all participants needed to be physically healthy, with no major medical conditions and score ≥ 27 in Mini Mental State Examination (MMSE); (2) those with current use of prescription medications or with a history of head injury or neurological illness were excluded; (3) those who met criteria of current or history of Axis I disorders according to the Structured Clinical Interview for DSM-IV (First et al., 1996) or reported current use of illicit substances or tested positive for cocaine, methamphetamine, opioids, marijuana, barbiturates, or benzodiazepines were excluded. Post-screening, we invited 62 volunteers for study participation. Among the 62, 2 were excluded because of MMSE score < 27, suggesting cognitive impairment (Salis et al., 2023), 4 could not complete the MR scan, and 5 with insufficient number of events in each fMRI conditions (see Section 2.4). Thus, we presented the data from 51 healthy adults (23 women) 22 to 80 years of age (Table 1). These participants were assessed with the State–Trait Anxiety Inventory (STAI) (Spielberger, 1989). The STAI state score ranged from 20 to 63 with a mean ± SD of 32.80 ± 10.54 in the current sample. Men and women did not differ in STAI state score (*t* = 1.33, *p* = 0.189), and differed marginally in age (*t* = 2.00, *p* = 0.050). The Human Investigation Committee at Yale School of Medicine approved the study procedures. All participants signed an informed consent prior to the study.

**Table 1:** Demographic information.

	All (*n* = 51)	Age < 60 years (*n* = 24)	Age ≥ 60 years (*n* = 27)	*t*-value/*χ*^2^-value, *p*-value
Age (years)	52.88 ± 18.53	35.29 ± 10.58	68.52 ± 4.70	14.77, <0.001
Sex (M/F)	28/23	10/14	18/9	3.21, 0.073
Education (years)	16.06 ± 2.69	15.92 ± 3.12	16.18 ± 2.30	0.35, 0.726
Race (C/B/A)	27/27/7	9/9/6	18/8/1	6.47, 0.039
STAI	32.80 ± 10.54	38.37 ± 10.57	27.85 ± 7.78	4.07, <0.001
MMSE	28.74 ± 0.89	28.92 ± 0.77	28.59 ± 0.97	1.31, 0.197
LM-II	12.23 ± 3.38	13.12 ± 3.23	11.44 ± 3.38	1.81, 0.077
BDI	7.53 ± 8.49	9.83 ± 9.45	5.48 ± 7.11	1.87, 0.067

*t*-Value/*χ*^2^-value/*p*-value represent differences between the two age-groups (age < 60; age ≥ 60 years), M, male; F, female; C, Caucasian; B, African American (Black); A, Asian; STAI, State–Trait Anxiety Inventory; MMSE, Mini Mental State Examination; LM-II, Logical Memory-II; BDI, Beck Depression Inventory.

### 2.2 Behavioral paradigm and performance metrics

**Figure 1:**
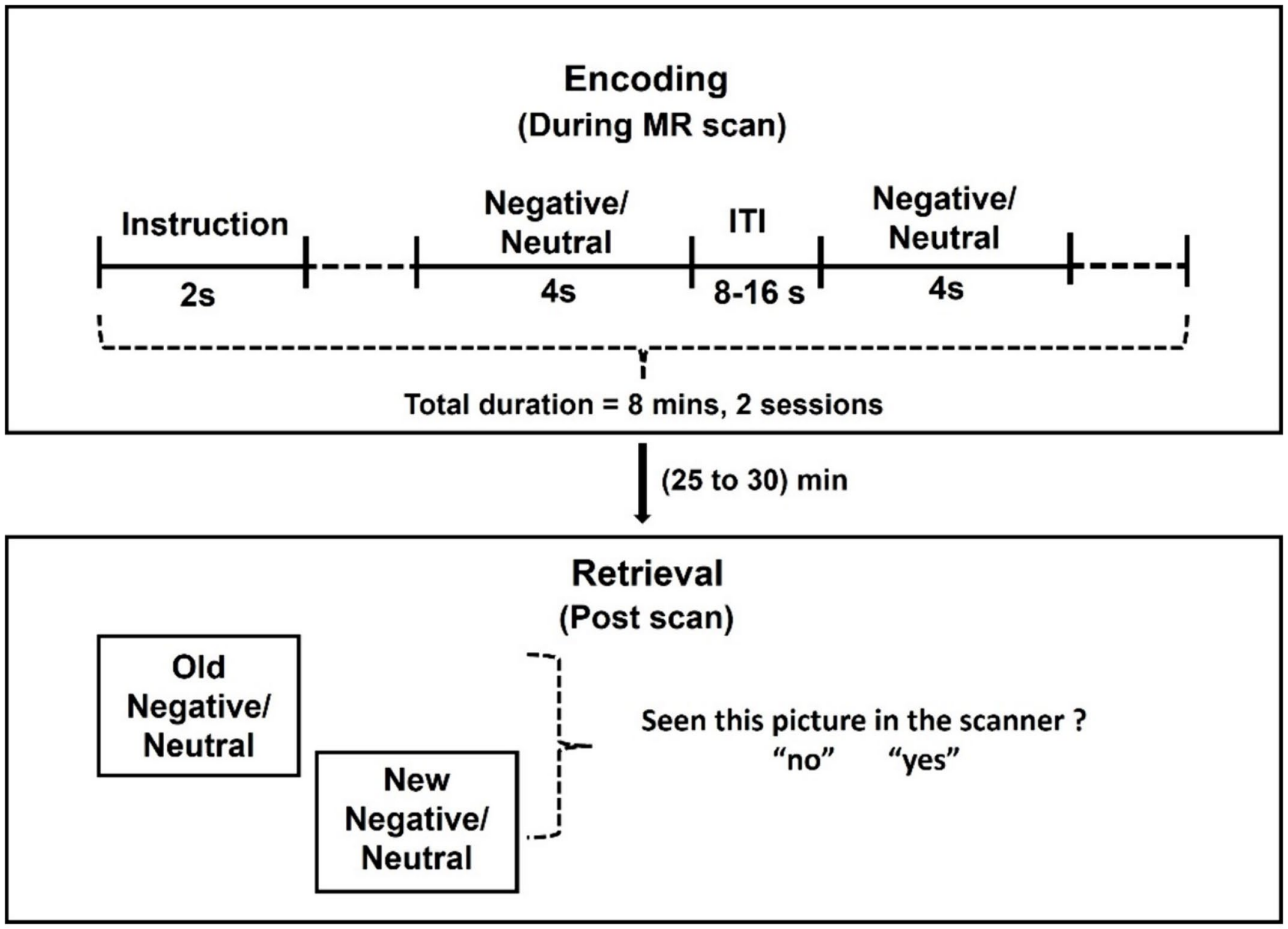
Behavioral paradigm. Forty emotional and 40 neutral pictures were obtained from the International Affective Picture System (IAPS) image set and presented in pseudorandomized order for 4 s each with an intertrial interval (ITI: blank screen) of a variable duration, ranging between 8 and 16 s to provide jitter. The total task duration was approximately 8 min. We presented the task in two sessions, each with a different set of images. Following the MR scan, participants were shown the same along with an equal number of new images and asked whether the images were presented before in the scanner or not.

We scanned participants during encoding of negative emotional and neutral pictures selected from the International Affective Picture System (IAPS) image set (Figure 1). Prior to MRI and again at the beginning of task during MRI, participants were instructed to remember the pictures and that they would be tested for how well they did after the scan. Forty negative emotional (normative valence = 2.72 ± 0.83; normative arousal = 5.67 ± 0.73) and 40 neutral (normative valence = 5.26 ± 0.89; normative arousal = 3.83 ± 0.83) pictures were presented in pseudorandomized order each for 4 s, with an intertrial interval (blank screen) 8 to 16 s in duration to provide jittering of the stimuli. The total task duration was approximately 8 min. We presented the task in two sessions, each with a different set of pictures.

The task was presented using a Dell Desktop (Windows 10; NVIDIA GeForce GT 1030, resolution: 1024 × 768; active signal resolution: 1024 × 768; refresh rate: 60 Hz) with Presentation Software (version 22.1 Build 04.30.21 by Neurobehavioral Systems, Inc.). The stimuli were projected onto an Optoma projector positioned at the front of the magnet bore opening, approximately 150 cm from the subject’s eyes. Subjects viewed this display through an inverted mirror placed on the head coil, positioned directly above their eyes.

Following the MR scan, participants were presented a picture one at a time from the entire set of pictures shown, interleaved with an equal number of new pictures (new negative picture normative valence = 2.72 ± 0.83, new negative picture normative arousal = 5.67 ± 0.73; new neutral picture normative valence = 5.34 ± 1.11, new neutral picture normative arousal = 3.86 ± 0.82) never shown during the MR scan, and queried whether the pictures were presented before. Participants answered yes or no to each picture.

According to signal detection theory (SDT) (Yu et al., 2018), the four possible outcomes for each trial/picture were: hits, misses, false alarm (FA), and correct rejection (CR). A hit and miss each represent an accurate and failed recognition that the picture was presented during the MR scan. An FA is false recognition of a picture never presented as presented and CR is correct recognition that such a picture was not presented. We computed the probability of hit rate (HR) and false alarm rate (FAR) as follows: P(HR) = *n*(hit)/[*n*(hit) + *n*(miss)]; P(FAR) = *n*(FA)/[*n*(FA) + *n*(CR)]. The sensitivity (*d’*) and bias were calculated as *d’* = Z_HR_ − Z_FAR_; bias = Z_FAR,_ where Z_HR_ and Z_FAR_ are the Z-score transforms, based on the normal distribution, of the P(HR) and P(FAR). We computed the Z-scores on the basis of negative and neutral trials combined, so the *d’* and bias (Z_FAR_) could be contrasted between negative and neutral trials in statistical analyses.

### 2.3 Imaging protocol and data pre-processing

Brain images were collected using multiband imaging with a 3-Tesla MR scanner (Siemens Trio, Erlangen, Germany). Conventional T1-weighted spin echo sagittal anatomical images were acquired for slice localization. Anatomical 3D MPRAGE image were next obtained with spin echo imaging in the axial plane parallel to the AC–PC line with TR = 1900 ms, TE = 2.52 ms, bandwidth = 170 Hz/pixel, field of view = 250 × 250 mm, matrix = 256 × 256, 176 slices with slice thickness = 1 mm and no gap. Functional, blood oxygen level-dependent (BOLD) signals were acquired with a single-shot gradient echoplanar imaging (EPI) sequence in two-sessions. Fifty-one axial slices parallel to the AC–PC line covering the whole brain were acquired with TR = 1,000 ms, TE = 30 ms, bandwidth = 2,290 Hz/pixel, flip angle = 62°, field of view = 210 × 210 mm, matrix = 84 × 84, 51 slices with slice thickness = 2.5 mm and no gap, 480 volumes, and multiband acceleration factor = 3. Images from the first ten TRs at the beginning of each scan were discarded to ensure that only BOLD signals in steady-state equilibrium between RF pulsing and relaxation were included in data analyses.

Data were analyzed with Statistical Parametric Mapping (SPM12, Welcome Department of Imaging Neuroscience, University College London, United States), following our published routines (Chaudhary et al., 2023b). Images of each individual subject were first realigned (motion corrected) and corrected for slice timing. A mean functional image volume was constructed for each subject per run from the realigned image volumes. These mean images were co-registered with the high-resolution structural image and segmented for normalization with affine registration followed by nonlinear transformation. The normalization parameters determined for the structure volume were then applied to the corresponding functional image volumes for each subject. The resampled voxel size is 2.5 × 2.5 × 2.5 mm^3^. Finally, the images were smoothed with a Gaussian kernel of 8 mm at Full Width at Half Maximum.

### 2.4 Imaging data: group analyses

We separated the negative and neutral trials according to the answers – “yes, I have seen the image” or “no, I have not seen the image” – to post-scan queries and categorized trials as negative-correct, negative-incorrect, neutral-correct, and neutral-incorrect. A statistical analytical design was constructed for each individual subject, using a general linear model (GLM) with the onsets of the event for each trial convolved with a canonical hemodynamic response function (HRF) and with the temporal derivatives of the canonical HRF and entered as regressors in the model (Friston et al., 1994). Realignment parameters in all six dimensions were also entered in the model. Serial autocorrelation caused by aliased cardiovascular and respiratory effects was corrected by a first-degree autoregressive or AR (1) model. The GLM estimated the component of variance explained by each of the regressors.

Following previous studies of memory encoding (Kensinger and Schacter, 2008; Spaniol et al., 2009; St Jacques et al., 2009; Fischer et al., 2010; Clemens et al., 2015; Dennis and Turney, 2018), we contrasted (negative-correct – negative-incorrect) versus (neutral-correct – neutral-incorrect) to identify the neural correlates specific to successful encoding of negative versus neutral emotional images. This contrast allows assessment of a direct association between brain-activity and performance specific to negative emotion memory (Dolcos et al., 2012). All subjects had 7 or more events in each of the 4 conditions (negative-correct, negative-incorrect, neutral-correct – neutral-incorrect), ensuring a sufficient number of events in each condition to observe a significant effect (Fischer et al., 2010).

To identify age-related and anxiety-related correlates, we performed a whole-brain linear regression of the contrast “(negative-correct – negative-incorrect) – (neutral-correct – neutral-incorrect)” on age and STAI state anxiety score, respectively. We evaluated the results at voxel *p* < 0.005, uncorrected in combination with cluster *p* < 0.05, FWE-corrected. For the clusters that showed a significant correlation with age, we checked for the correlation of their activities (β’s) with STAI state score. Likewise, for the clusters that showed a significant correlation with STAI state score, we checked for the correlation of their β’s with age.

### 2.5 Mediation analyses

For the clusters with activities of “(negative-correct – negative-incorrect) – (neutral-correct – neutral-incorrect)” showing a significant correlation with both age and STAI state score, we performed mediation analyses to characterize the inter-relationships of these variables (See Results). We performed mediation analyses as with our previous studies (Chaudhary et al., 2023a,b) and described in the Supplemental methods. The analysis was performed with package ‘Medsem’ (Mehmetoglu, 2018) in Stata: Statistical software for data science (StataCorp LLC).

## 3 Results

### 3.1 Age, behavioral performance, and clinical characteristics

**Figure 2:**
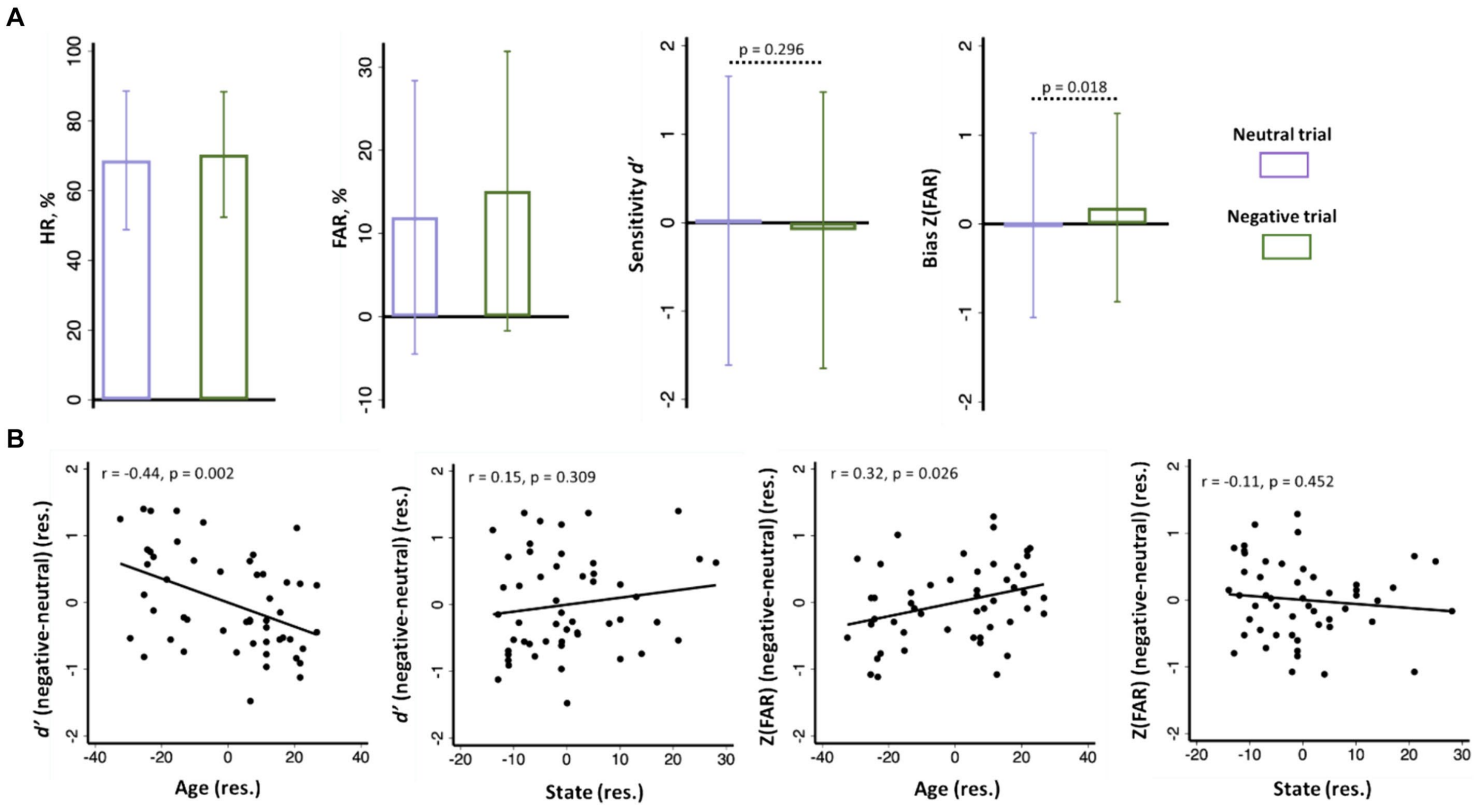
**(A)** Hit rate or HR (%), false alarm rate or FAR (%), sensitivity *d’*, and bias Z(FAR) of negative and neutral images shown in bar plots of mean ± SD; **(B)** *d’* (negative - neutral) and Z(FAR) (negative - neutral) were significantly correlated with age, but not with anxiety, with sex as covariate. Note: the data points in scatterplots represent values of the residuals (res.).

The mean ± standard deviation (SD) of hit rate (HR), false alarm rate (FAR), sensitivity *d’*, and bias Z(FAR) are shown in Figure 2A. Participants showed higher bias or Z(FAR) (*t* = 2.43, *p* = 0.019, paired *t*-test) and indistinguishable sensitivity *d’* (*t* = 1.05, *p* = 0.296, paired t test) in recognizing negative versus neutral emotional images.

Across subjects, the differences in Z(FAR) [negative Z(FAR) – neutral Z(FAR); *r* = 0.31, *p* = 0.025] and in *d’* (negative *d’* – neutral *d’*; *r* = −0.44, *p* = 0.001) in recalling negative versus neutral images were both significantly correlated with age, with sex as a covariate (Figure 2B). Thus, age was associated with higher bias but lower sensitivity in recognizing negative relative to neutral images.

Participants showed a STAI state score of 32.8 ± 10.5 (mean ± SD). STAI state score was significantly and negatively correlated with age, with sex as a covariate (*r* = −0.52, *p* < 0.001). STAI state score was not significantly correlated with the difference in *d’* (negative *d’* – neutral *d’*; *r* = 0.15, *p* = 0.309) or with the difference in Z(FAR) [negative Z(FAR) − negative Z(FAR); *r* = −0.11, *p* = 0.452] in a linear regression with sex as a covariate (Figure 2B). Thus, individual variation in state anxiety was not significantly associated with the bias or sensitivity in recognizing negative emotional relative to neutral images.

### 3.2 Neural correlates of emotional memory

**Figure 3:**
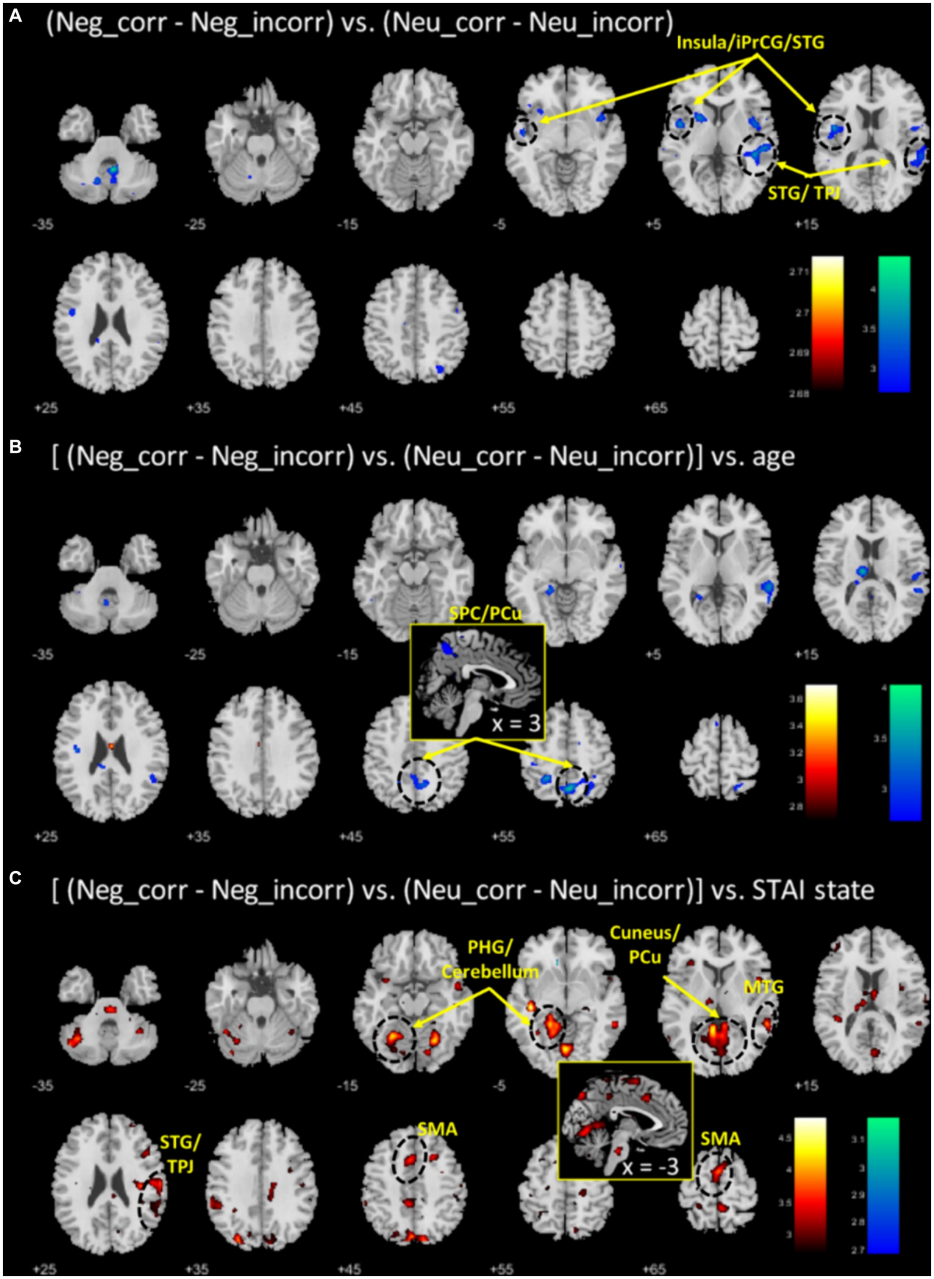
**(A)** Regional correlates of “negative_correct - negative_incorrect” (neg_corr - neg_incorr) versus “neutral_correct - neutral_incorrect” (neu_corr - neu_incorr). Color bars show voxel T value of (neg_corr - neg_incorr) < (neu_corr - neu_incorr); the reverse contrast did not show any significant voxels. **(B,C)** Each shows the regional correlates of age and anxiety regression on (neg_corr - neg_incorr) – (neu_corr - neu_incorr). Warm/cool color bar each shows the T values of voxels in positive/negative correlation. Voxel *p* < 0.005, uncorrected.

To identify the correlates specific to negative emotional memory, we contrasted “negative-correct – negative-incorrect” with “neutral-correct – neutral-incorrect” and showed the regional activities in Figure 3A. Two clusters each of the left insula/inferior precentral gyrus/superior temporal gyrus (INS/iPrCG/STG; x = −43, y = −1, z = 18, Z = 3.95, 335 voxels) and right superior temporal gyrus/temporo-parietal junction (STG/TPJ; x = 43, y = −34, z = 6, Z = 3.86, 424 voxels) showed lower activation during correctly versus incorrectly recognized emotional images, as compared to the same of neutral images. At the same threshold, no regional activities were significant for the reverse contrast. The mean brain activations for all relevant contrasts are presented in the Supplementary Figures S1, S2 and Supplementary Tables S1, S2.

### 3.3 Age- and anxiety-related neural correlates of emotional memory

**Figure 4:**
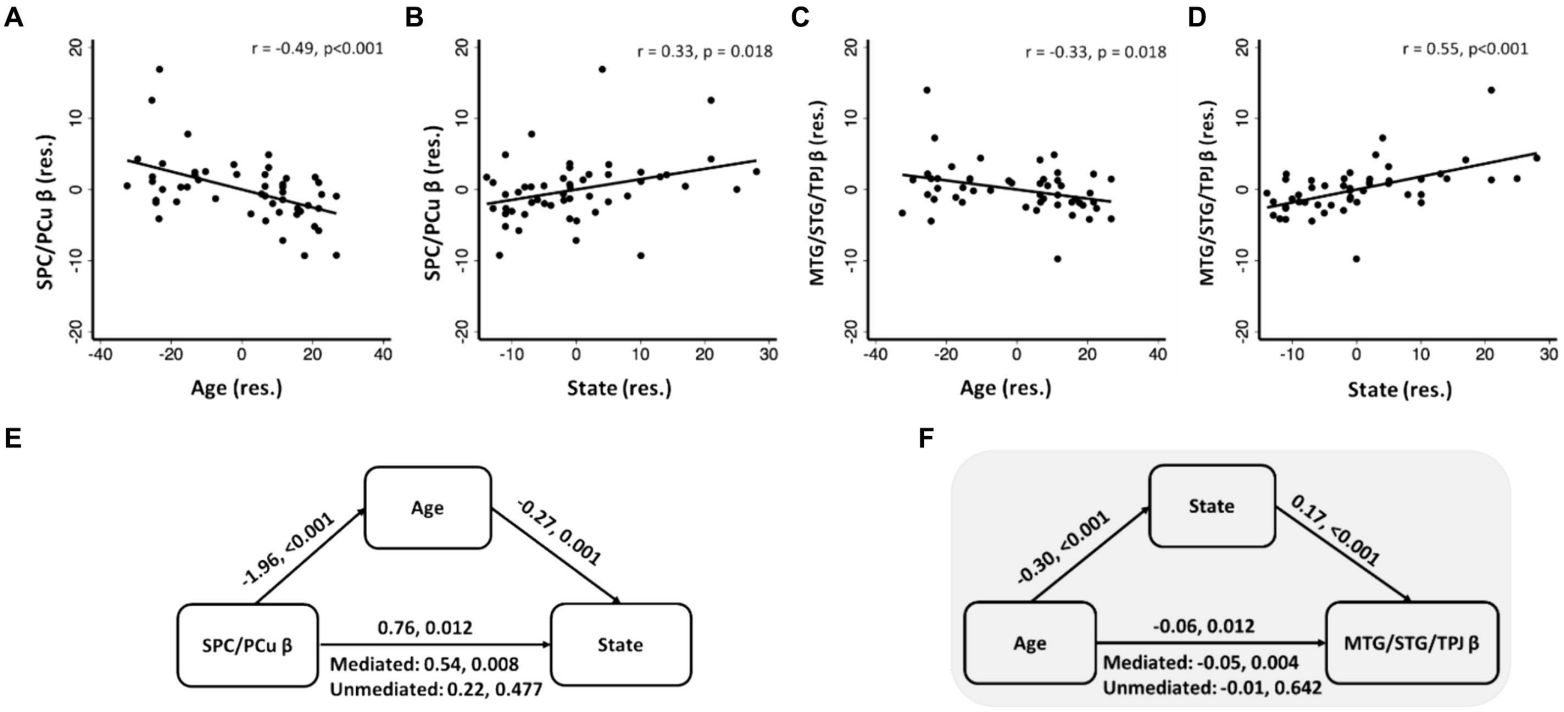
SPC/PCu β **(A,B)** and MTG/STG/TPJ β **(C,D)** were significantly correlated with age and anxiety, with sex as covariate; **(E,F)** significant mediation models with SPC/PCu β or MTG/STG/TPJ β, age and state. Scatterplots present data in residuals (res.). The two values noted for each mediation path represent the coefficient and *p* value, respectively. Shaded panel highlighted the only mediation model that was significant when age was considered solely as an independent variable.

To identify age-related correlates of negative emotional memory, we performed a whole brain regression of the contrast “(negative_correct - negative_incorrect) – (neutral_correct - neutral_incorrect)” on age, with sex as a covariate, and showed the regional activities in Figure 3B. Only the cluster that included the superior parietal cortex and precuneus (SPC/PCu) met cluster *p* < 0.05, FWE-corrected (x = 3, y = −59, z = 53, Z = 3.72, 437 voxels). We extracted the β value of “(negative_correct - negative_incorrect) – (neutral_correct - neutral_incorrect)” of the SPC/PCu cluster for testing in mediation analysis, and correlation with clinical variables. As expected, the β value of right SPC/PCu was significantly and negatively correlated with age, with sex as a covariate (*r* = −0.49, *p* < 0.001; Figure 4A). The β value of the SPC/PCu cluster was also significantly and positively correlated with anxiety score (*r* = 0.33, *p* = 0.018; Figure 4B), and with *d’*(negative-neutral) (*r* = 0.35, *p* = 0.013), with sex as a covariate. We did not test for correlation with the reporting bias Z(FAR) as these did not represent neural activities during memory encoding.

To identify anxiety-related correlates of negative emotional memory, we performed a whole brain regression of the difference in sensitivity “(negative_correct - negative_incorrect) – (neutral_correct - neutral_incorrect)” on STAI score, with sex as a covariate, and showed the regional activities in Figure 3C. Four clusters met cluster *p* < 0.05, FWE-corrected: the left parahippocampal gyrus and part of the cerebellar cortex (x = −23, y = −56, z = −12, Z = 4.15, 1,541 voxels), right middle/superior temporal gyri and temporo-parietal junction (MTG/STG/TPJ; x = 58, y = −44, z = 1, Z = 4.19, 626 voxels), cuneus and precuneus (x = −3, y = −79, z = 48, Z = 3.83, 472 voxels), and the supplementary and pre-supplementary motor areas (x = −13, y = 12, z = 51, Z = 4.00, 418 voxels). Of these four clusters, only the right MTG/STG/TPJ showed a β contrast in significant correlation with age, with sex as a covariate (*r* = −0.33, *p* = 0.018; *p*’s > 0.07 for other clusters) (Figure 4C). Right MTG/STG/TPJ β (*r* = 0.32, *p* = 0.022) also correlated positively with *d’* (negative-neutral) with sex as a covariate.

### 3.4 Mediation analysis of age, anxiety, and neural correlates of negative emotional memory

As presented in the previous section, we observed 3-way pair-wise correlations among age, STAI score, and the β “(negative_correct - neutral_correct) – (negative_incorrect - neutral_incorrect)” of the SPC/PCu identified from age regression (Figures 4A,B) and of the right MTG/STG/TPJ identified from STAI score regression (Figures 4C,D). We ran mediation analyses with sex as a covariate to further investigate the inter-relationship amongst these clinical and neural measures. For each cluster, we tested all six models and evaluated the results at a corrected *p* < 0.05/6 = 0.0083. It’s important to note that, while all six mediation models were tested, our focus was specifically on models with age as the independent variable. For SPC/PCu, a significant mediation was noted in model β → age → anxiety (Figure 4E), whereas for the right MTG/STG/TPJ, mediation analyses showed a significant model with age → anxiety → β value (Figure 4F), both with sex as a covariate. The statistics of all other models are shown in Supplementary Tables S3A,B. If we considered solely models where age served as an independent variable, only the model age → anxiety → MTG/STG/TPJ β was significant.

## 4 Discussion

We observed that age was associated with both higher reporting bias but lower sensitivity in recognizing negative versus neutral images. That is, older versus young people were more likely to have incorrectly reported recognizing negative versus neutral images, although they fared worse in encoding and/or retrieving memory. Age was also negatively correlated with anxiety. However, across participants STAI anxiety score was not correlated with either reporting bias or sensitivity in recognizing negative versus neutral images. These findings suggest that, with correction for reporting bias, age is associated both with impaired encoding/retrieval of negative versus neutral emotional images and with diminished anxiety. However, this impairment in negative emotional memory cannot explain the age-related changes in anxiety.

Whole-brain regression showed that activity of the right superior parietal cortex/precuneus (SPC/PCu) and right middle/superior temporal gyri (MTG/STG) and temporoparietal junction (TPJ) during successful encoding of negative versus neutral images decreased and increased with age and anxiety, respectively. Further, anxiety mediated the relationship between age and MTG/STG/TPJ activity, suggesting that the temporo-parietal cortical activities reflect the consequence of an anxiety state. We discussed the main findings below.

### 4.1 Negative emotional memory

Negative emotions can enhance both the accuracy (Kensinger, 2007; Mather and Sutherland, 2011) and/or subjective feelings of vividness or confidence for retrieved memory (Dougal and Rotello, 2007; Phelps and Sharot, 2008). Previous studies observed higher sensitivity in recalling negative versus neutral images (Anderson et al., 2006; Rimmele et al., 2011; Xie and Zhang, 2017; Yeh et al., 2021). Here, we did not observe a significant difference in sensitivity in recalling negative versus neutral images. On the other hand, we observed higher reporting bias in recalling negative versus neutral images. Indeed, while recollection was more accurate for negative than neutral scenes (Anderson et al., 2006; Yeh et al., 2021), Rimmele et al. noted enhanced subjective feeling of remembering negative versus neutral scenes without actually remembering the scene details (Rimmele et al., 2011). The other study noted item memory as enhanced under negative versus neutral conditions was also associated with elevated subjective feelings of remembering the conditions (Xie and Zhang, 2017). Together, these findings suggest the critical importance of accounting for reporting bias in evaluating the accuracy of recall.

### 4.2 Age and negative emotional memory

We noted age-associated reduction in sensitivity *d’* in recognizing negative emotions, consistent with some (Charles et al., 2003; Leigland et al., 2004; Kensinger and Schacter, 2008; Leal et al., 2015; Stam et al., 2022) but not other (Kensinger et al., 2002, 2007a,b; Fischer et al., 2010) studies. Notably, Fischer et al., 2010 did not correct for bias when evaluating response accuracy. With bias accounted for, the memory performance would trend similarly to our findings (Hit-false alarm rate for negative emotional (fearful face): young = 0.35 ± 0.15, old = 0.24 ± 0.19; and for neutral face: young = 0.26 ± 0.19, old = 0.17 ± 0.14) (Fischer et al., 2010), suggesting an increase in false alarms with age (as we discuss next). In addition to reporting bias, a number of other factors may influence age-related differences in encoding accuracy. For instance, Kensinger et al., noted that in studies where participants passively viewed but were not instructed to memorize the pictures, young and older adults performed equally in memory (Kensinger et al., 2002, 2007a). However, in studies that required encoding, younger adults performed better than older adults (Kensinger et al., 2007b). Thus, the differences in study design and the role of active versus passive engagement in encoding may contribute to the observed discrepancies in age-related memory performance across studies. Further, although age was significantly associated with lower levels of anxiety, STAI scores were not significantly correlated with *d’*. This finding suggests that age-related reduction in negative emotional memory is unlikely to account for age-related reduction in anxiety. Together with the earlier reports, the present findings are consistent with age-related positivity in emotional memory, which, however, cannot explain the changes in state anxiety in this sample.

We found that age was associated with higher reporting bias during recall of negative versus neutral images. Compared to young adults, older adults have objectively less accurate event memory, but over-estimated event vividness (Folville et al., 2020, 2022). Age-related cognitive changes may have imposed on older people to rely more on familiarity-based monitoring, which along with reduced inhibition of irrelevant information, induces false memories (Devitt and Schacter, 2016). However, how emotion affects such an age-related tendency toward false memories is still not clear. Gallo et al., noted false memory to be higher in response to emotional than to neutral pictures, with older versus young adults showing higher frequency of false memory of emotional pictures (Gallo et al., 2009). In another study with emotional/neutral words presented during encoding and new emotional/neutral lure words during retrieval, both young and older adults performed equally and showed less false memory of emotional versus neutral words (Kensinger and Corkin, 2004). A possible explanation for the discrepant findings concerns the stimuli, with studies using images but not those using words showing an age effect, although depending on the context in which lure words were presented, word stimuli could elicit false memories (Garry and Wade, 2005; Smith and Hunt, 2020).

### 4.3 Anxiety and negative emotional memory

We did not observe a significant association between sensitivity or reporting bias with anxiety, in-line with earlier reports that did not find any differences in recall in high versus low anxiety individuals (Wolfensberger et al., 2008; van Tol et al., 2012), and in contrast with other reports of better recall of negative versus neutral emotional stimuli in high anxiety individuals (Reidy and Richards, 1997; Lee and Fernandes, 2018; Yu et al., 2018; Hakamata et al., 2022; note that only Lee and Fearnandes and Yu et al. controlled for reporting bias in evaluating accuracy in recall). Exposure to stress prior to encoding enhances encoding of emotional versus neutral stimuli (Payne et al., 2007; Hoscheidt et al., 2014). On the other hand, negative emotional states can disrupt memories; a recent meta-analysis showed that negative emotions elevated recall failures in a working memory task and that the effects were moderated by participants’ self-reported severity of negative life experiences (Xie et al., 2022). Other studies with image pairs-negative and neutral images presented in pairs of both neutral, both negative, or mixed; noted varied impacts of context on memory (Kensinger and Schacter, 2006; Bisby et al., 2016; Ritchey et al., 2019). While we cannot currently explain the absence of a behavioral association between anxiety and memory, future studies may explore these explanations. Further, despite the lack of a behavioral association, we observed a correlation between regional encoding of negative emotion and anxiety, consistent with previous studies (Wolfensberger et al., 2008; van Tol et al., 2012). This is probably because encoding may be more sensitive to anxiety, while retrieval may share a complex relationship with anxiety (Gagnon and Wagner, 2016).

### 4.4 Age- and anxiety- related neural correlates of negative emotional memory

Age and anxiety correlated negatively and positively, respectively, with right MTG/STG/TPJ activity during “(negative correct > incorrect) – (neutral correct > incorrect)” trials, and anxiety mediated the association between age and MTG/STG/TPJ activity. The MTG has been implicated in successful encoding of emotional memory (Murty et al., 2010; Dahlgren et al., 2020; Riegel et al., 2022). The STG is involved in social cognition (Allison et al., 2000) and emotion processing (Tseng et al., 2016; Liu et al., 2022), and the TPJ in differentiating old and new memory in memory updating (Simon et al., 2017). Previous studies have noted either no age-related changes (Kensinger and Schacter, 2008) or reduced MTG (hippocampal) activity (St Jacques et al., 2009; Fischer et al., 2010) during correct versus incorrect negative versus neutral encoding in older adults. Our findings are consistent with the study that used scenes (St Jacques et al., 2009), but not those that used objects (Kensinger and Schacter, 2008; Addis et al., 2010). This discrepancy may be due to different stimuli engaging different brain regions during processing (Preckel et al., 2019). In anxiety disorders, previous literature supported structural/functional deficits of the temporal lobe. For instance, relative to healthy participants, individuals (15–55 years age) with anxiety disorders demonstrated lower left MTG/STG volumes (van Tol et al., 2010). Another study of young adolescents noted greater right MTG volume in individuals scoring higher in social anxiety (Wang et al., 2021). A meta-analysis of structural and resting-state functional connectivity identified altered amygdala-MTG/STG connectivity in anxiety disorders (Cao et al., 2021). Exposure to stressful versus neutral stimuli led to higher magnetoencephalographic activity in the medial temporal lobe, especially during memory encoding (Heinbockel et al., 2021). Collectively, diminished anxiety with age may dampen negative emotion encoding in MTG.

Age and anxiety also correlated negatively and positively, respectively, with SPC/PCu activity in the contrast of “(negative correct > incorrect) – (neutral correct > incorrect)” trials. Along with the MTG, the parietal cortex is implicated in threat detection and encoding as well as hypervigilance and excessive monitoring of environment in the pathophysiology of anxiety (Carlsson et al., 2006). Higher SPC activity during anticipation of an unpredictable aversive event was associated with subjective anxiety scores in healthy participants (Hasler et al., 2007). Individuals with social anxiety disorder demonstrated higher SPC activity in processing threatening versus neutral visual scenes (Goldin et al., 2009; Heitmann et al., 2016). As the hub of default mode network and in support of self-reflection and self-focused thinking (Cavanna and Trimble, 2006), the precuneus is also implicated in anxiety. The precuneus showed elevated amplitude of low-frequency fluctuations in individuals with social anxiety as compared to controls (Yuan et al., 2018). An earlier meta-analysis noted precuneus hyperactivity during processing of negative versus neutral (and positive) emotional stimuli in individuals with post-traumatic stress disorder (Etkin and Wager, 2007). Importantly, a broad literature highlighted age-related changes in precuneus activity in a wide range of behavioral tasks. For instance, while watching a video and responding to each meaningful event, which may be important for episodic memory retrieval, the PCu amongst other posterior medial brain regions showed higher activity and this increase in activity became less prominent with age (Reagh et al., 2020). Our findings are thus consistent with this literature. Further, the SPC/Pcu activity was positively correlated with both anxiety score and negative versus neutral *d’* (sensitivity), though mediation models failed to substantiate the relationship amongst [age, SPC/PCu β, and *d’*] (data not shown) or amongst [age, SPC/PCu, and anxiety] (model in support of SPC/PCu β → age → anxiety; Figure 4E). A larger sample size is needed to revisit this issue.

### 4.5 Reduced temporoparietal cortical and insula activity in negative emotional memory

Averaging across all participants, we observed lower left insula/iPrCG/STG and right STG/TPJ responses to successfully versus unsuccessfully remembered negative versus neutral images. The insula supports visceral/interoceptive, sensorimotor, and homeostatic processing as well as emotional awareness and response selection (Nieuwenhuys, 2012). In the context of memory encoding, a recent animal study noted enhanced consolidation of object memory by the basolateral amygdala through suppression of insular cortical activity, suggesting a possible role of the insula in allocating resources for memory (Chen et al., 2022). The STG is involved in social cognition (Allison et al., 2000) and emotion processing (Tseng et al., 2016; Liu et al., 2022), and the TPJ in differentiating old and new memory in memory updating (Simon et al., 2017). A previous study noted greater STG activation during successful versus unsuccessful encoding of emotional (negative + positive) versus neutral images (Mickley Steinmetz and Kensinger, 2009). A meta-analysis observed enhanced STG activity during the encoding and PrCG activity during the retrieval of emotional items or neutral items encoded in an emotional context (Dahlgren et al., 2020). In our study, we observed an overall reduction in activity, which may be attributed to the inclusion of subjects across the lifespan. Notably, a previous study that similarly encompassed subjects across the lifespan reported reduced activity in the PrCG and STG during the processing of emotional compared to neutral items (Daley et al., 2020). Nevertheless, further investigation of this issue is warranted.

### 4.6 Limitations and conclusions of the study

A number of limitations need to be considered. First, we focused here only on negative and neutral emotional memory. Investigating the neural processes of positive emotion processing will better our understanding of the mechanisms of “positivity effect” and age- related reduction in anxiety. It is also important to note that, whereas the great majority of studies employed the contrast “negative correct - neutral correct” to identify the correlates of memory, we considered incorrect trials in the contrast to control for the perceptual processes, as with several earlier studies (Kensinger and Schacter, 2008; Spaniol et al., 2009; St Jacques et al., 2009; Fischer et al., 2010; Clemens et al., 2015; Dennis and Turney, 2018). The specific contrasts used would dictate the results and may account for the discrepancy between the current and some of the previous findings. Second, we did not scan subjects during the retrieval phase. Previous studies have shown common and distinct regions involved in encoding and retrieval (Prince et al., 2005; Dahlgren et al., 2020), both of which may be altered by age (Srokova et al., 2022) and anxiety (Wolfensberger et al., 2008). Imaging data collected during recall would help in elucidating the neural correlates of reporting bias and how neural processes of encoding and recall may be related. Third, the STAI state score ranged from 20 to 63 in the current sample. Participants with higher state score are needed to better understand the effects of anxiety as well as the impact of age on anxiety, with the caveat that most people with the highest state scores have a clinical diagnosis of anxiety and depressive disorders and typically receive mediations for their conditions. Our sample size is moderate; studies of a larger sample may allow us to investigate the mediation models with an adequate power and sex differences in the roles of emotional memory in supporting age-related reduction in anxiety. Finally, not all older adults display reduced anxiety. Increased anxiety is evident in older adults with subjective cognitive decline (SCD) (Liew, 2020), mild cognitive impairment (MCI) (Devier et al., 2009; Li et al., 2014; Klimova et al., 2015), and Alzheimer’s Disease (AD) (Devier et al., 2009). Along with anxiety, these cohorts also display emotion processing dysfunction. For instance, individuals with AD display impaired identification of negative facial emotions, inconsistent emotional memory benefit (Brueckner and Moritz, 2009; Klein-Koerkamp et al., 2012; Mistridis et al., 2013; Cárdenas et al., 2021), and inconsistent positivity effect (Goodkind et al., 2015; Zhang et al., 2015). People with MCI exhibit impaired negative emotion recognition in people with MCI (Elferink et al., 2015; Morellini et al., 2022); and those with SCD show altered facial emotion processing(Perez et al., 2022) and emotion dysregulation (Mah et al., 2021). Further, anxiety represents a risk factor of cognitive decline in the elderly (Pietrzak et al., 2012; Santabárbara et al., 2020; Gracia-García et al., 2023). Thus, impaired emotion processing and anxiety may be linked to early cognitive changes. Our study cohort includes typically healthy, cognitively intact participants without severe anxiety. Thus, the findings represent healthy aging. Future studies should target older adults with high anxiety and cognitive impairments.

In conclusion, age is associated with lower levels of anxiety and reduced sensitivity but higher reporting bias in recognizing negative emotions. The right middle temporal gyrus/temporal parietal junction/superior temporal gyrus (MTG/TPJ/STG) show activity (β) during “negative correct – incorrect” – “neutral correct – incorrect” trials each in positive and negative correlation with anxiety and with age. Mediation analyses supported a complete mediation model of age → less anxiety → less MTG/TPJ/STG. Together, the findings demonstrate age-related changes in negative emotional memory and how age-related reduction in anxiety is reflected in diminished temporal–parietal cortical activities during encoding of negative emotional memory.

## Data availability statement

The original contributions presented in the study are included in the article/Supplementary material, further inquiries can be directed to the corresponding author.

## Ethics statement

The studies involving humans were approved by The Human Investigation Committee at Yale School of Medicine. The studies were conducted in accordance with the local legislation and institutional requirements. The participants provided their written informed consent to participate in this study.

## Author contributions

SC: Formal analysis, Project administration, Writing – original draft. SZ: Data curation, Formal analysis, Methodology, Writing – review & editing. YC: Formal analysis, Writing – review & editing. JD: Conceptualization, Writing – review & editing. HC: Conceptualization, Funding acquisition, Writing – review & editing. C-SL: Conceptualization, Funding acquisition, Supervision, Validation, Writing – review & editing.
