# Supplementary material for: Age-related reduction in anxiety and neural encoding of negative emotional memory
Source: Front Aging Neurosci. 2024 Jul 3;16:1375435. doi: 10.3389/fnagi.2024.1375435 (PMC11252031; doi:10.3389/fnagi.2024.1375435)
Supplement: Supplementary file 1 [file Data_Sheet_1.docx]

**Age-related reduction in anxiety and neural activities encoding negative emotional memory**

Shefali Chaudhary, Sheng Zhang, Yu Chen, Jacqueline C. Dominguez, Herta H. Chao, Chiang-Shan R. Li

**Supplemental methods: mediation analysis**

***Mediation analysis***

To examine the interrelationships of BOLD responses (beta estimates) of identified regions, age, GP, and AUDIT score, we conducted mediation analyses using a single-mediator model. The methods were detailed in our previous work (Wang et al., 2020; Zhornitsky et al., 2019). Briefly, in a mediation analysis, the relation between the independent variable X and dependent variable Y; that is, X → Y is tested to determine whether it is significantly mediated by a variable M. The mediation test is performed using the following three regression equations:

Y=i_1_+cX+e_1_

Y=i_2_+c′X+bM+e_2_

M=i_3_+aX+e_3_

where a represents X → M, b represents M → Y (controlling for X), c' represents X → Y (controlling for M), and c represents X → Y. The constants i_1_, i_2_, i_3_ are the intercepts, and e_1_, e_2_, e_3_ are the residual errors. In the literature, a, b, c, and c' are commonly referred to as “path coefficients,” Variable M is said to be a mediator of connection X → Y, if (c – c'), which is mathematically equivalent to the product of the paths a × b, is significantly different from zero. If (c – c') is different from zero and the paths a and b are significant, one concludes that X → Y is mediated by M. In addition, if path c' is not significant, it indicates that there is no direct connection from X to Y and that X → Y is completely mediated by M. The analysis was performed with package ‘Medsem’ in STATA (Mehmetoglu, 2018). To test the significance of the mediation effect, we used the bootstrapping method as it is generally considered advantageous to the Sobel test (Zhao et al., 2010).


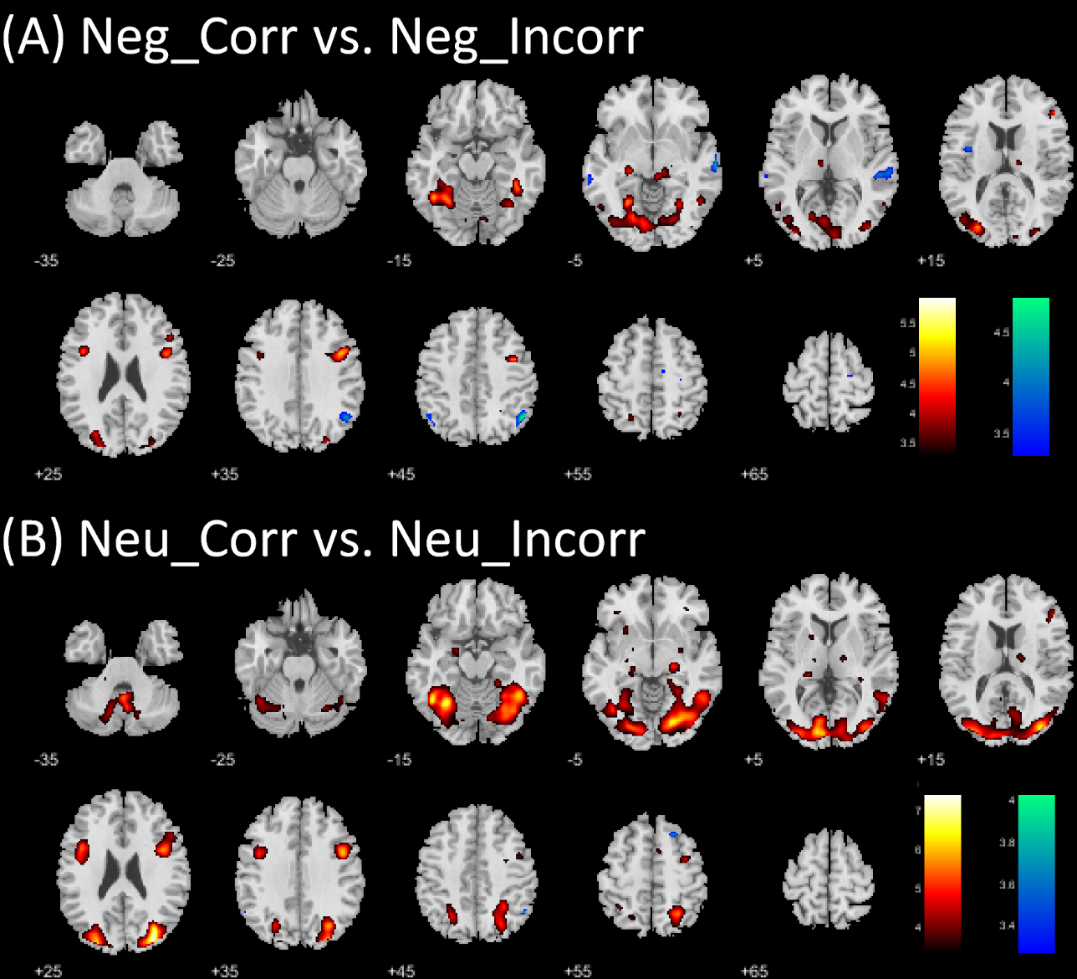


**Supplementary Figure S1.** Regional activations shown in one-sample t test of negative-correct vs. negative-incorrect and of neutral-correct vs. neutral-incorrect trials. Color bars reflect voxel T values (warm: neg-correct > neg-incorrect; neu-correct > neu-incorrect; cool: neg-correct < neg-incorrect; neu-correct < neu-incorrect). **Supplementary Table S2** summarizes the clusters that met cluster p<0.05, FWE-corrected.

**Supplementary Table S1A**. Whole-brain activations during negative correct vs negative incorrect condition in one-sample t-test.

| Cluster size (k) | x | y | z | Z-value | Region |
| --- | --- | --- | --- | --- | --- |
| *Negative Correct > Negative Incorrect* | | | | | |
| 2575 | -25 | -56 | -10 | 4.80 | L FG |
|  | 40 | -39 | -15 | 4.49 | R FG |
|  | -43 | -51 | -17 | 4.46 | L FG |
| 280 | 43 | 7 | 31 | 5.11 | R PCG |
|  | 40 | 2 | 41 | 4.83 |  |
|  | 28 | 7 | 38 | 3.76 | R MFG |
| *Negative Incorrect > Negative Correct* | | | | | |
| 137 | 48 | -59 | 48 | 4.35 | R AG |
| 165 | 65 | -21 | -2 | 4.29 | R STG |
|  | 53 | -29 | 3 | 3.90 |  |

Note: L: left hemisphere, R: right hemisphere, FG: fusiform gyrus, PCG: precentral gyrus, MFG: middle frontal gyrus, AG: angular gyrus, STG: superior temporal gyrus

**Supplementary Table S1B**. Whole-brain activations during neutral-correct vs. neutral-incorrect trials: one-sample t-test.

| Cluster size (k) | x | y | z | Z-value | Region |
| --- | --- | --- | --- | --- | --- |
| *Neutral Correct > Neutral Incorrect* | | | | | |
| 7131 | 30 | -84 | 28 | 6.05 | R SOG |
|  | -30 | -59 | -12 | 6.00 | L FG |
|  | -45 | -54 | -15 | 5.80 | L ITG |
| 493 | 45 | 2 | 31 | 5.86 | R PCG |
|  | 45 | 24 | 21 | 4.26 | R IFG |
|  | 50 | 32 | 13 | 4.14 |  |
| 84 | 23 | -21 | -2 | 5.08 | R Thalamus |
| 317 | -40 | 9 | 26 | 4.84 | L IFG |
|  | -40 | 4 | 33 | 4.57 | L PCG |
| 89 | -29 | -6 | -10 | 4.68 | L Putamen |
| 42 | -33 | 32 | -12 | 4.53 | L IFG |
|  | -38 | 32 | 1 | 3.15 |  |
| 133 | -20 | -29 | 1 | 4.36 | L Thalamus |
|  | -15 | -14 | 8 | 3.88 |  |
| *Neutral Incorrect > Neutral correct* | | | | | |
| None | | | | | |

Note: L: left hemisphere, R: right hemisphere, SOG: superior occipital gyrus, FG: fusiform gyrus, ITG: inferior temporal gyrus, IFG: inferior frontal gyrus, PCG: precentral gyrus

**
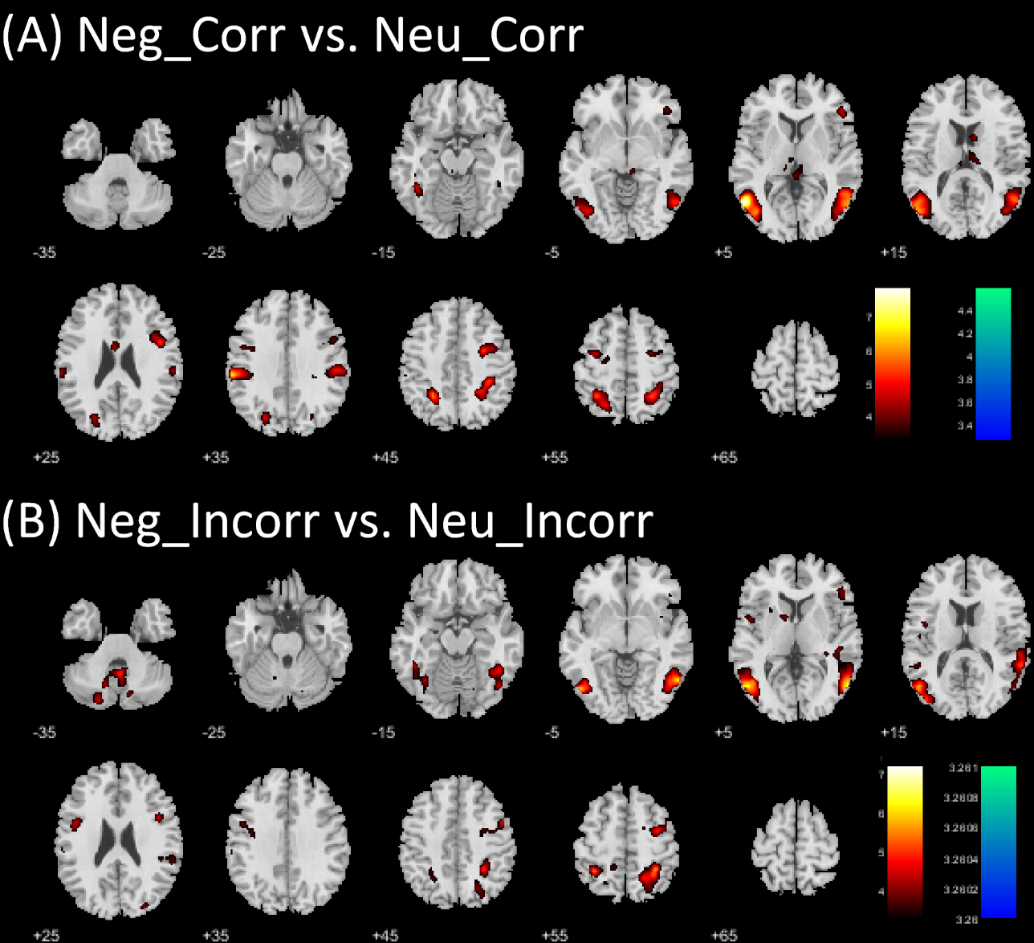
**

**Supplementary Figure S2.** Regional activations of one-sample t test of (A) negative-correct vs. neutral-correct and (B) negative-incorrect vs. neutral-incorrect trials. **Supplementary Table S3** summarizes the clusters with cluster p<0.05 FWE-corrected.

**Supplementary Table S2A**. Whole-brain activations during negative correct vs neutral correct condition in one-sample t-test.

| Cluster size (k) | x | y | z | Z-value | Region |
| --- | --- | --- | --- | --- | --- |
| *Negative Correct > Neutral Correct:* | | | | | |
| 845 | -55 | -61 | 3 | 6.32 | L MTG |
|  | -53 | -71 | 11 | 5.95 | L MOG |
| 692 | -58 | -29 | 36 | 5.76 | L SMG |
|  | -25 | -51 | 46 | 5.12 | L SPL |
|  | -33 | -51 | 58 | 4.93 |  |
| 717 | 55 | -64 | 1 | 5.63 | R MTG |
|  | 58 | -61 | 11 | 5.32 |  |
|  | 53 | -54 | 3 | 5.07 |  |
| 443 | 35 | -36 | 46 | 4.94 | R SPL |
|  | 28 | -49 | 53 | 4.86 |  |
| 207 | 45 | 7 | 23 | 4.86 | R IFG |
| 204 | 28 | -4 | 46 | 4.84 | R PCG |
|  | 38 | 2 | 43 | 4.27 | R MFG |
| 214 | 53 | -24 | 33 | 4.54 | R SMG |
|  | 60 | -26 | 31 | 4.52 |  |
| 140 | -25 | -74 | 31 | 4.52 | L SOG |
| 123 | 48 | 34 | 3 | 4.17 | R IFG |
|  | 43 | 31 | -7 | 3.62 |  |
|  | 50 | 24 | 11 | 3.30 |  |
| 155 | 8 | -16 | 13 | 3.94 | R Thalamus |
|  | -5 | -24 | 13 | 3.57 | L Thalamus |
|  | 0 | -16 | 8 | 3.53 |  |
| *Neutral Correct > Negative Correct* | | | | | |
| None |  |  |  |  |  |

Note: L: left hemisphere, R: right hemisphere, MTG: middle temporal gyrus, MOG: middle occipital gyrus, SMG: supramarginal gyrus, SPL: superior parietal lobule, IFG: inferior frontal gyrus, PCG: precentral gyrus, MFG: middle frontal gyrus, SMG: supramarginal gyrus, SOG: superior occipital gyrus

**Supplementary Table S2B**. Whole-brain activations during negative incorrect vs neutral incorrect condition in one-sample t-test.

| Cluster size (k) | x | y | z | Z-value | Region |
| --- | --- | --- | --- | --- | --- |
| *Negative Incorrect > Neutral Incorrect* | | | | | |
| 915 | -50 | -66 | 11 | 5.94 | L MTG |
|  | -48 | -69 | -7 | 5.45 | L IOG |
|  | -40 | -81 | 16 | 4.52 | L MOG |
| 1186 | 55 | -61 | 1 | 5.72 | R MTG |
|  | 45 | -66 | -10 | 5.14 | R ITG |
|  | 60 | -44 | 13 | 4.67 | R MTG |
| 209 | 3 | -54 | -32 | 5.31 | R Cerebellum |
|  | -13 | -64 | -35 | 3.90 | L Cerebellum |
|  | -13 | -59 | -27 | 3.70 |  |
| 593 | 28 | -4 | 51 | 5.20 | R MFG |
|  | 20 | -54 | 53 | 4.59 | R SPL |
|  | 23 | -66 | 46 | 3.73 |  |
| 192 | -35 | -49 | 56 | 4.84 | L SPL |
|  | -23 | -59 | 51 | 3.66 |  |
| 221 | 35 | -6 | 51 | 4.70 | R PCG |
|  | 48 | 4 | 46 | 4.27 |  |
|  | 25 | -9 | 43 | 3.46 |  |
| 60 | 43 | 9 | 26 | 4.18 | R IFG |
| 41 | 13 | -76 | -40 | 4.16 | R Cerebellum |
| 55 | -20 | -79 | -35 | 4.03 | L Cerebellum |
| 208 | -45 | 4 | 26 | 4.03 | L PCG |
|  | -43 | -1 | 16 | 4.15 | L IFG |
| *Neutral Incorrect > Negative Incorrect* | | | | | |
| None |  |  |  |  |  |

Note: MTG: middle temporal gyrus, IOG: inferior occipital gyrus, MOG: middle occipital gyrus, ITG: inferior temporal gyrus, SPL: superior parietal lobule, MFG: middle frontal gyrus

**Table S3A.** Mediation model β and p-values (X/M/Y: independent/mediating/dependent variable with sex as covariate)

| X | M | Y | β, p- value | | | | |
| --- | --- | --- | --- | --- | --- | --- | --- |
|  |  |  | X 🡪 M | M 🡪 Y | X 🡪 Y | Direct  X 🡪 Y | Indirect  X 🡪 Y |
| Age | SPC/PCu β | State | -0.13, <0.001 | 0.22,  0.477 | -0.30,  <0.001 | -0.27,  <0.001 | -0.03,  0.484 |
| Age | State | SPC/PCu β | -0.39,  <0.001 | 0.04,  0.477 | -0.13,  <0.001 | -0.11,  0.002 | -0.01,  0.483 |
| SPC/PCu β | Age | State | -1.96,  <0.001 | -0.27,  0.001 | 0.76,  0.012 | 0.22,  0.477 | 0.54,  0.008* |
| State | Age | SPC/PCu β | -0.89,  <0.001 | -0.11,  0.002 | 0.14,  0.012 | 0.04,  0.477 | 0.10,  0.011 |
| State | SPC/PCu β | Age | 0.14,  0.012 | -1.43,  0.002 | -0.89,  <0.001 | -0.69,  0.001 | -0.21,  0.049 |
| SPC/PCu β | State | Age | 0.76,  0.012 | -0.69,  0.001 | -1.96,  <0.001 | -1.43,  0.002 | -0.52,  0.042 |

*p<0.0083

**Table S3B.** Mediation model β and p-values (X/M/Y: independent/mediating/dependent variable with sex as covariate)

| X | M | Y | β, p- value | | | | |
| --- | --- | --- | --- | --- | --- | --- | --- |
|  |  |  | X 🡪 M | M 🡪 Y | X 🡪 Y | Direct  X 🡪 Y | Indirect  X 🡪 Y |
| Age | MTG/STG/TPJ β | State | -0.06, 0.012 | 1.28,  <0.001 | -0.30,  <0.001 | -0.22,  0.001 | -0.08,  0.036 |
| Age | State | MTG/STG/TPJ β | -0.30,  <0.001 | 0.17,  <0.001 | -0.06,  0.012 | -0.01,  0.642 | -0.05,  0.004* |
| MTG/STG/TPJ β | Age | State | -1.73,  0.012 | -0.22,  0.001 | 1.66,  <0.001 | 1.28,  <0.001 | 0.38,  0.043 |
| State | Age | MTG/STG/TPJ β | -0.89,  <0.001 | -0.01,  0.642 | 0.18,  <0.001 | 0.17,  <0.001 | 0.01,  0.644 |
| State | MTG/STG/TPJ β | Age | 0.18,  <0.001 | -0.34,  0.642 | -0.89,  <0.001 | -0.83,  0.001 | -0.06,  0.644 |
| MTG/STG/TPJ β | State | Age | 1.66,  <0.001 | -0.83,  0.001 | -1.73,  0.012 | -0.34,  0.642 | -1.38,  0.006* |

*p<0.0083

**References:**

Mehmetoglu, M. (2018). medsem: a Stata package for statistical mediation analysis. *International Journal of Computational Economics and Econometrics*, *8*, 63. https://doi.org/10.1504/IJCEE.2018.10007883

Wang, W., Zhornitsky, S., Le, T. M., Zhang, S., & Li, C.-S. R. (2020). Heart Rate Variability, Cue-Evoked Ventromedial Prefrontal Cortical Response, and Problem Alcohol Use in Adult Drinkers. *Biological Psychiatry. Cognitive Neuroscience and Neuroimaging*, *5*(6), 619–628. https://doi.org/10.1016/j.bpsc.2019.12.013

Zhao, X., Lynch, J., & Chen, Q. (2010). Reconsidering Baron and Kenny: Myths and Truths About Mediation Analysis. *Journal of Consumer Research*, *37*, 197–206. https://doi.org/10.1086/651257

Zhornitsky, S., Zhang, S., Ide, J. S., Chao, H. H., Wang, W., Le, T. M., Leeman, R. F., Bi, J., Krystal, J. H., & Li, C.-S. R. (2019). Alcohol Expectancy and Cerebral Responses to Cue-Elicited Craving in Adult Nondependent Drinkers. *Biological Psychiatry. Cognitive Neuroscience and Neuroimaging*, *4*(5), 493–504. https://doi.org/10.1016/j.bpsc.2018.11.012
